# Mechanical Properties of a Chiral Cellular Structure with Semicircular Beams

**DOI:** 10.3390/ma14112887

**Published:** 2021-05-27

**Authors:** Yalei Bai, Tong Zhao, Chengxu Yuan, Weidong Liu, Haichao Zhang, Lei Yang, Chongmin She

**Affiliations:** 1 College of Aerospace Engineering, Nanjing University of Aeronautics and Astronautics, Nanjing 210016, China; tzhao@nuaa.edu.cn; 2 College of Energy and Electrical Engineering, Hohai University, Nanjing 211100, China; 1805050212@hhu.edu.cn (C.Y.); liuweidong@hhu.edu.cn (W.L.); 1705060106@hhu.edu.cn (H.Z.); 3 North Information Control Research Academy Group Co. Ltd., Nanjing 211153, China; yanglei55866@gmail.com

**Keywords:** chiral structure, semicircular beam, tensile modulus, shear modulus, coupling effect

## Abstract

Compliant cellular structures are good candidates for morphing applications. This paper proposes a novel chiral cellular structure composed of circular beams with great elastic properties and potential for morphing. The tensile and shear elastic properties of the structure are studied through theoretical derivations and then verified by finite element analysis. Results show that this novel chiral structure exhibits extremely low in-plane tensile and shear moduli, which are many orders of magnitude lower than that of the raw material. The structure also shows tensile–shear and shear–tensile coupling effects that cannot be ignored. The tensile and shear properties of the structure can provide a reference for employing this structure in engineering applications.

## 1. Introduction

Cellular structures have been widely investigated because of their outstanding mechanical properties, such as their light weight, high rigidity, and high strength [1,2,3]. They have been broadly applied in aerospace, navigation, and civil engineering. Conventional studies mainly concentrate on enhancing the stiffness and strength of cellular structures as the core of sandwich plates by constructing structures with stretching-dominated rods [4,5,6,7,8,9,10]. However, these cellular structures with their high stiffnesses are unsuitable in situations where compliant structures are required.

In recent years, compliant cellular structures with low stiffness for morphing and energy absorption applications have attracted increasing attention. Compliant cellular structures can be employed as underlying supports for structures in newly developed morphing aircrafts [11,12], morphing wind turbines [13,14,15], morphing automobiles [16], soft robots [17,18], and biomedical stents [19]. The conventional honeycomb structure with slender walls can be regarded as a kind of compliant cellular structure whose elastic properties were comprehensively studied by Gibson and Ashby [20]. As a variant of the classical honeycomb, the re-entrant honeycomb was investigated by Masters and Evans [21]. A combination of the two structures above was proposed by Olympio and Gandhi for morphing skin applications [22]. Various compliant cellular structures have been successively proposed, and their elastic properties studied [23,24,25,26,27,28,29,30,31]. These studies reveal that cellular structures with bending-dominated beams exhibit significant compliance.

Among the numerous compliant cellular structures, the chiral cellular structure has a fascinating layout (it cannot mirror itself by in-plane translation or rotation) and unique properties and has received considerable attention from scientists all over the world. Lakes et al. first proposed a chiral cellular structure with a hexagonal unit of six bendable ligaments and rotatable central circles [32,33]. Then, tri- and tetra-chiral cellular structures and their variants and combinations were studied by Alderson et al. [34,35,36,37,38,39,40,41,42]. These studies show that the chiral cellular structures with great compliance are very suitable for applications in precision engineering [43,44] and morphing aircraft engineering [45,46].

The most studied chiral cellular structures are composed of central circles connected with bendable ligaments. In 2000, Smith et al. proposed a new type of chiral cellular structure, which is constructed by four connecting V-type beams [47]. Bahaloo and Liu et al. studied the in-plane elastic properties of the structure through micropolar theory and the energy method, respectively [48,49]. As a new kind of chiral cellular structure, the literature on the new configurations or properties of such structures is limited, restricting the development and application of this new type of chiral cellular structure.

In the studies of elastic properties of compliant cellular structures, analytical analysis is often employed to obtain theoretical models of structure’s elastic constants [50], and finite element analysis is usually utilized as verification for the theoretical models. In the theoretical modeling of the elastic constants, Euler–Bernoulli beam model considering the internal bending moment and axial force has been widely used [51,52]. In the finite element analysis, various novel finite element models have been proposed by scientists in exploring mechanics of structures [53,54]. A classic software named ANSYS, has been extensively accepted for finite element modeling and analysis in engineering fields. It is capable of analyzing slender to moderately thick beam structures [55], and capable of handling 1D to 3D models reliably [56].

In this paper, a novel configuration of this new kind of chiral cellular structure with circular beams is proposed. Different from structures reported in literature, the unit cell of the chiral structure proposed in this study is constructed by four semi-circular beams evenly connecting at the central point. The proposed structure shows a simpler layout than conventional chiral structures with rotatable central circles, and exhibits lower in-plane stiffness with great potential for morphing application. The feasible region of the geometric parameters of the structure is studied by numerical analysis. Then, the structural elastic properties are theoretically investigated and verified through finite element analysis in ANSYS. The effects of the geometric parameters on the elastic properties, including the in-plane tensile modulus, the shear modulus, and the tensile–shear and shear–tensile coupling effects are comprehensively discussed. Finally, conclusions are drawn as guidelines for employing the chiral cellular structure in morphing applications.

## 2. Geometry of the Structure

**Figure 1:**
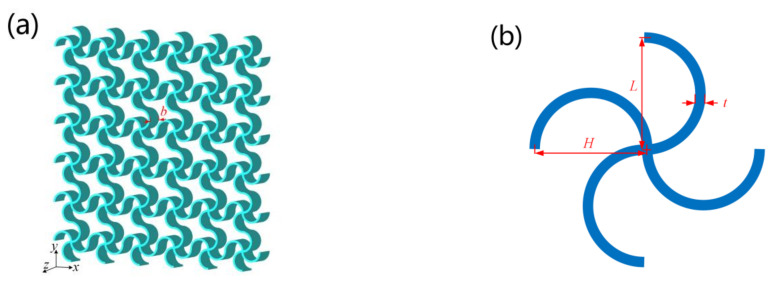
Geometry of the structure: (**a**) 3D structure and (**b**) 2D unit cell.

Figure 1 shows the three-dimensional and the two-dimensional layouts of the structure. The unit cell is a chiral structure composed of four semicircular beams connected at the central point. The diameters of the semicircles along the x- and y-directions are denoted as *L* and *H*, respectively. The thickness of the beams is denoted as *t*. The gauge thickness along the z-direction is denoted as *b*. In this paper, nondimensionalized parameters α (=*H/L*), β (=*t/L*), and *γ* (=*b/L*) are employed in the modeling of the elastic properties of the structure.

**Figure 2:**
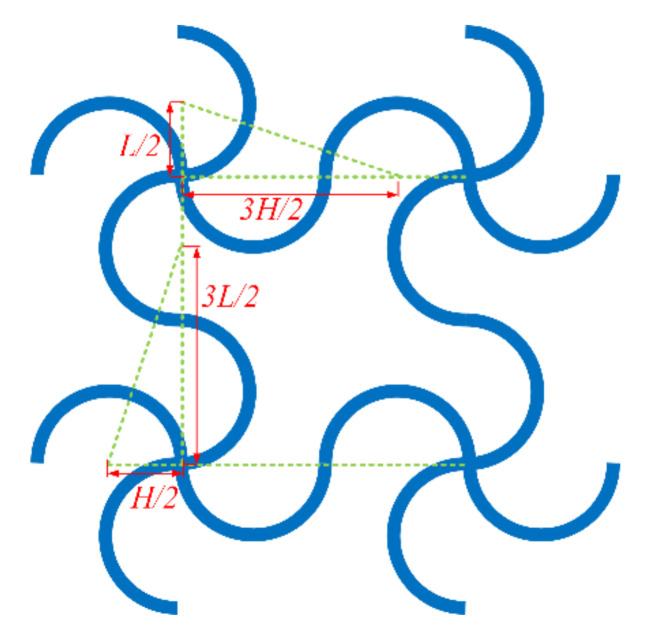
Overlapping analysis.

To avoid overlapping circular beams, the geometric parameters must obey certain constraints. As shown in Figure 2, the parameters should satisfy the following inequality to avoid overlapping along the x-direction:

(1) $$\sqrt{{\left(\frac{3H}{2}\right)}^{2}+{\left(\frac{L}{2}\right)}^{2}}>\frac{H}{2}+\frac{L}{2}+t$$

Meanwhile, the parameters should satisfy the following inequality to avoid overlapping along the y-direction:

(2) $$\sqrt{{\left(\frac{H}{2}\right)}^{2}+{\left(\frac{3L}{2}\right)}^{2}}>\frac{H}{2}+\frac{L}{2}+t$$

Thus, by considering *L* > *t* and *H* > *t*, the nondimensionalized parameters α and β must satisfy the following constraints:

(3) $$\left\{\begin{array}{c} 4\alpha^{2}-2\alpha \beta -2\beta^{2}-\alpha -2\beta >0\\ 4-2\alpha \beta -2\beta^{2}-\alpha -2\beta >0\\ \beta <1\\ \beta <\alpha \end{array}\right.$$

**Figure 3:**
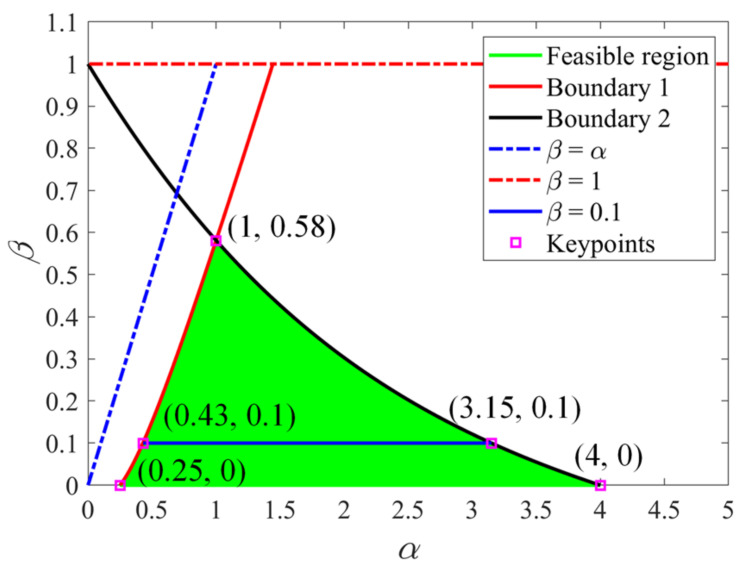
Feasible region of geometric parameters.

Then, the feasible region of parameters α and β are plotted as shown in Figure 3 (the area in green) according to Equation (3). The key points of the feasible region are highlighted in Figure 3. The feasible range for parameter α is (0.25, 4). The feasible range for parameter β increases from 0 to 0.58 when α increases from 0.25 to 1 and then decreases from 0.58 to 0 when α increases from 1 to 4. In this study, the assumption of β ≤ 0.1 is employed in the analysis to apply Euler beam theory in the derivations of the elastic properties of the structure. Hence, the corresponding range of parameter α is (0.43, 3.15), as shown in Figure 3. 

## 3. Modeling of the Elastic Properties

### 3.1. Tensile Properties in the X-Direction

**Figure 4:**
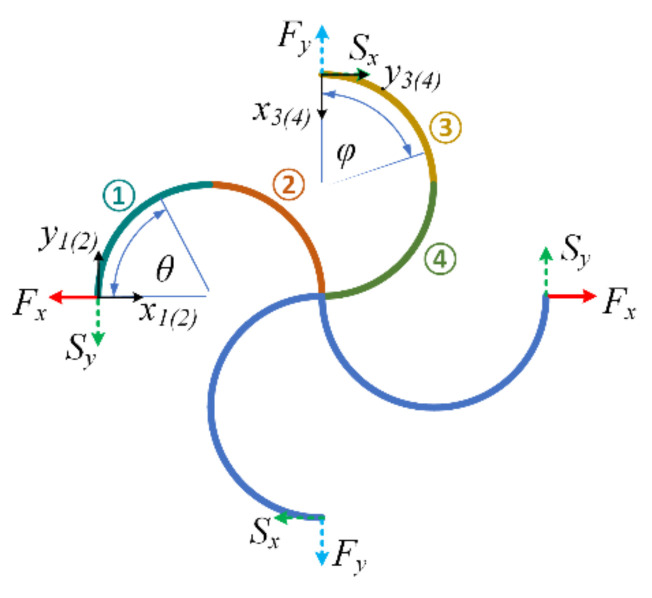
Unit cell under tensile loading in the x-direction.

According to the coordinate systems shown in Figure 4, the profile of the circular beams can be written as follows:

(4) $$\left\{\begin{array}{c} x_{i}=\frac{H}{2}\left(1-\cos\theta \right),\;y_{i}=\frac{H}{2}\sin\theta ,\;i=1,\;2\\ x_{j}=\frac{L}{2}\left(1-\cos\varphi \right),\;y_{j}=\frac{L}{2}\sin\varphi ,\;j=3,\;4 \end{array}\right.$$

When the periodic chiral structure undergoes a far-field tension along the x-direction, the four end points of the unit cell are moment-free because of the chiral layout of the structure. No tensile force exists along the y-direction at the top and bottom end points. Furthermore, no shear load is imposed on the four end points. Thus, only a pair of tensile forces (marked in red in Figure 4) acts on the left and right end points of the unit cell. 

In the modeling, all the tensile and shear loads are considered in the derivation of the strain energy function of the unit cell to facilitate the derivation of the subsequent shear properties. By considering the internal moments and axial forces, the equation of the unit cell’s strain energy can be written as follows:

(5) $$U=2\left\{\begin{array}{c} \int_{0}^{\frac{\pi }{2}}\frac{{\left(S_{y}\sin\left(\frac{\pi }{2}-\theta \right)+F_{x}\cos\left(\frac{\pi }{2}-\theta \right)\right)}^{2}}{2EA}\frac{H}{2}d\theta +\int_{\frac{\pi }{2}}^{\pi }\frac{{\left(S_{y}\sin\left(\theta -\frac{\pi }{2}\right)-F_{x}\cos\left(\theta -\frac{\pi }{2}\right)\right)}^{2}}{2EA}\frac{H}{2}d\theta \\ +\int_{0}^{\frac{\pi }{2}}\frac{{\left(S_{x}\sin\left(\frac{\pi }{2}-\varphi \right)-F_{y}\cos\left(\frac{\pi }{2}-\varphi \right)\right)}^{2}}{2EA}\frac{L}{2}d\varphi +\int_{\frac{\pi }{2}}^{\pi }\frac{{\left(S_{x}\sin\left(\varphi -\frac{\pi }{2}\right)+F_{y}\cos\left(\varphi -\frac{\pi }{2}\right)\right)}^{2}}{2EA}\frac{L}{2}d\varphi \\ +\int_{0}^{\frac{\pi }{2}}\frac{{\left(S_{y}x_{1}-F_{x}y_{1}\right)}^{2}}{2EI}\frac{H}{2}d\theta +\int_{\frac{\pi }{2}}^{\pi }\frac{{\left(S_{y}x_{2}-F_{x}y_{2}\right)}^{2}}{2EI}\frac{H}{2}d\theta \\ +\int_{0}^{\frac{\pi }{2}}\frac{{\left(S_{x}x_{3}+F_{y}y_{3}\right)}^{2}}{2EI}\frac{L}{2}d\varphi +\int_{\frac{\pi }{2}}^{\pi }\frac{{\left(S_{x}x_{4}+F_{y}y_{4}\right)}^{2}}{2EI}\frac{L}{2}d\varphi \end{array}\right.$$

where *E* is the Young’s modulus of the raw material, *A* = *bt* is the cross-sectional area of the beams, and *I* = *bt*^3^/12 is the cross-sectional inertia moment of the beams.

Thus, the deformation of the unit cell along the x-direction under the tensile load can be obtained as follows:

(6) $$\delta_{x}={\left.\frac{\partial U}{\partial F_{x}}\right|}_{S_{x}=S_{y}=F_{y}=0}=\frac{\pi \alpha \left(3\alpha^{2}+\beta^{2}\right)}{2E\beta^{3}\gamma L}F_{x}$$

The effective tensile stress and strain can be expressed as follows:

(7) $$\left\{\begin{array}{c} \sigma_{x}=\frac{F_{x}}{2bL}\\ \varepsilon_{x}=\frac{\delta_{x}}{2H} \end{array}\right.$$

Then, the non-dimensional effective tensile modulus can be obtained as follows:

(8) $$\frac{E_{x}}{E}=\frac{2\beta^{3}}{\pi \left(3\alpha^{2}+\beta^{2}\right)}$$

The deformation of the unit cell along the y-direction under the tensile load in the x-direction can also be obtained as $\delta_{y}={\left.\frac{\partial U}{\partial F_{y}}\right|}_{S_{x}=S_{y}=F_{y}=0}=0$. Therefore, the effective Poisson’s ratio of the structure is $\nu_{xy}=0$.

The structure also exhibits shear deformation under the tensile load, and the deformation in the two in-plane directions can be obtained as follows:

(9) $$\left\{\begin{array}{c} \delta_{\varepsilon x}={\left.\frac{\partial U}{\partial S_{x}}\right|}_{S_{x}=S_{y}=F_{y}=0}=0\\ \delta_{\varepsilon y}={\left.\frac{\partial U}{\partial S_{y}}\right|}_{S_{x}=S_{y}=F_{y}=0}=-\frac{6\alpha^{3}}{E\beta^{3}\gamma L}F_{x} \end{array}\right.$$

Then, the shear strain under tensile load can be written as:

(10) $$\gamma_{\varepsilon }=\frac{\delta_{\varepsilon x}}{2L}+\frac{\delta_{\varepsilon y}}{2H}=-\frac{3\alpha^{2}}{E\beta^{3}\gamma L^{2}}F_{x}$$

Thus, the tensile–shear coupling effect can be expressed as the ratio of the shear strain to the tensile strain as follows:

(11) $$\frac{\gamma_{\varepsilon }}{\varepsilon_{x}}=-\frac{12\alpha^{2}}{\pi \left(3\alpha^{2}+\beta^{2}\right)}$$

In addition, the elastic properties of the structure in the y-direction can be obtained by substituting the parameters (1/α for α, β/α for β) into the corresponding equations because the structure exhibits a 90-degree rotational similarity.

### 3.2. Shear Properties in the X–Y Plane

**Figure 5:**
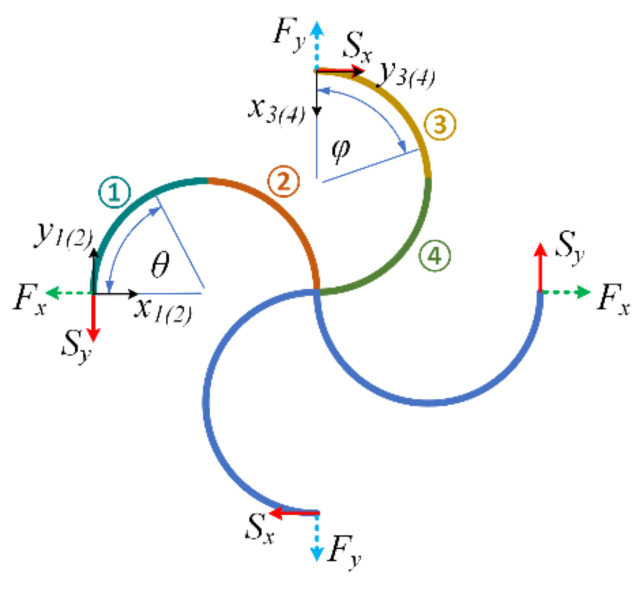
Unit cell under shear loading in the x–y plane.

When the periodic chiral structure undergoes a far-field shear stress, there is no moment or tensile force acting on the four end points of the unit cell. Only shear forces exist at the four end points. The shear forces are plotted in red in Figure 5.

The shear modulus can be obtained by taking the partial derivative of the strain energy function, which is determined using Equation (5) in Section 3.1. The shear deformations of the unit cell can be obtained as follows:

(12) $$\left\{\begin{array}{c} \delta_{sx}={\left.\frac{\partial U}{\partial S_{x}}\right|}_{F_{x}=F_{y}=0}=\frac{\pi \left(\beta^{2}+9\right)}{2E\beta^{3}\gamma L}S_{x}\\ \delta_{sy}={\left.\frac{\partial U}{\partial S_{y}}\right|}_{F_{x}=F_{y}=0}=\frac{\pi \alpha \left(9\alpha^{2}+\beta^{2}\right)}{2E\beta^{3}\gamma L}S_{y} \end{array}\right.$$

Then, the shear strain can be obtained as follows:

(13) $$\gamma_{xy}=\frac{\delta_{sx}}{2L}+\frac{\delta_{sy}}{2H}=\frac{\pi \left(9\alpha^{2}S_{y}+\beta^{2}S_{y}+\beta^{2}S_{x}+9S_{x}\right)}{4E\beta^{3}\gamma L^{2}}$$

Shear forces $S_{x}$ and $S_{y}$ can be written as follows:

(14) $$\left\{\begin{array}{c} S_{x}=2bH\tau_{xy}\\ S_{y}=2bL\tau_{xy} \end{array}\right.$$

The non-dimensional effective shear modulus can be obtained as follows:

(15) $$\frac{G_{xy}}{E}=\frac{\tau_{xy}}{E\gamma_{xy}}=\frac{2\beta^{3}}{\pi \left(\alpha \beta^{2}+9\alpha^{2}+9\alpha +\beta^{2}\right)}$$

Then the structure also exhibits tensile deformations when it undergoes in-plane shear loading. The tensile deformations along the two in-plane directions can be, respectively, obtained as follows:

(16) $$\left\{\begin{array}{c} \delta_{\gamma x}={\left.\frac{\partial U}{\partial F_{x}}\right|}_{F_{x}=F_{y}=0}=-\frac{6\alpha^{3}}{E\beta^{3}\gamma L}S_{y}\\ \delta_{\gamma y}={\left.\frac{\partial U}{\partial F_{y}}\right|}_{F_{x}=F_{y}=0}=\frac{6}{E\beta^{3}\gamma L}S_{x} \end{array}\right.$$

The tensile strains along the two in-plane directions can be written as follows:

(17) $$\left\{\begin{array}{c} \varepsilon_{\gamma x}=\frac{\delta_{\gamma x}}{2H}\\ \varepsilon_{\gamma y}=\frac{\delta_{\gamma y}}{2L} \end{array}\right.$$

Thus, the shear–tensile coupling effects can be described by the tensile strain to shear strain ratios as follows:

(18) $$\left\{\begin{array}{c} \frac{\varepsilon_{\gamma x}}{\gamma_{xy}}=-\frac{12\alpha^{2}}{\pi \left(\alpha \beta^{2}+9\alpha^{2}+9\alpha +\beta^{2}\right)}\\ \frac{\varepsilon_{\gamma y}}{\gamma_{xy}}=\frac{12\alpha }{\pi \left(\alpha \beta^{2}+9\alpha^{2}+9\alpha +\beta^{2}\right)} \end{array}\right.$$

## 4. Finite Element Analyses

**Figure 6:**
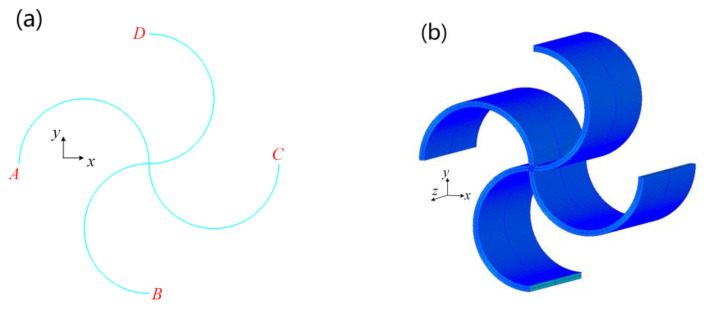
Finite element model: (**a**) 2D curve model and (**b**) 3D model.

Finite element analyses were performed in ANSYS (version 18.0, ANSYS Inc., Canonsburg, PA, USA). A parametric two-dimensional model of the unit cell was established, as shown in Figure 6a. The beam element (BEAM 189) possessing three nodes, with six degrees of freedom for each node, was applied to the curves to realize the 3D finite element model, as shown in Figure 6b. In the model, parameter *L* was set to 20 mm. Parameter α varied from 0.5 to 3 with a step of 0.1, while parameter β varied from 0.01 to 0.1 with a step of 0.01. Parameter *γ* was set to 1. The following base functions are employed in ANSYS:

(19) $$\begin{array}{c} N_{1}=\frac{1}{4}\left(1-\xi \right)\left(1-\eta \right)\\ N_{2}=\frac{1}{4}\left(1+\xi \right)\left(1-\eta \right)\\ N_{3}=\frac{1}{4}\left(1+\xi \right)\left(1+\eta \right)\\ N_{4}=\frac{1}{4}\left(1-\xi \right)\left(1+\eta \right) \end{array}$$

In the analyses, hexahedral mesh was employed to mesh the model. The element size was set to 0.1 mm after a convergence test. The Young’s modulus of the raw material was set to 2.6 Gpa, and the Poisson’s ratio was 0.3 based on the properties of a common engineering material, namely, polyformaldehyde resin.

The weak form was obtained without considering the body force load, and can be written as follows:

(20) $$\underset{V}{\int }\sigma_{ij}\delta e_{ij}dV=\underset{S}{\int }f_{i}^{S}\delta u_{i}dS$$

where $\sigma_{ij}$ is Cauchy stress component, $e_{ij}=\frac{1}{2}\left(\frac{\partial u_{i}}{\partial x_{j}}+\frac{\partial u_{j}}{\partial x_{i}}\right)$ is the deformation tensor, $u_{i}$ is the displacement, $x_{i}$ is the current coordinate, $f_{i}^{S}$ is the component of surface traction, V is the volume of the deformed body, and S is the surface of deformed body on which tractions are prescribed.

The boundary conditions for tensile loading in the x-direction are as follows:

(21) $$\left\{u\right\}=\left\{\begin{array}{c} \left\{0\right\}\;for\;node\left(s\right)\;at\;the\;left\;edge\;\\ \left\{\begin{array}{c} u_{xt}\\ 0 \end{array}\right\}\;for\;node\left(s\right)\;at\;the\;right\;edge \end{array}\right.$$

The boundary conditions for tensile loading in the x–y plane are as follows:

(22) $$\left\{u\right\}=\left\{\begin{array}{c} \left\{0\right\}\;for\;node\left(s\right)\;at\;the\;bottom\;edge\;\\ \left\{\begin{array}{c} u_{xs}\\ 0 \end{array}\right\}\;for\;node\left(s\right)\;at\;the\;upper\;edge \end{array}\right.$$

Periodic conditions were applied to the unit cell to realize the simulation of an infinite periodic structure. Table 1 lists the periodic and boundary conditions employed in the analyses. In the table, SYMM refers to the symmetric condition. The deformations of the unit cell were realized by imposing displacements (*ε_x_* = 0.05, *γ_xy_* = 0.05) on the end points. The effective stresses were calculated by the reaction forces. Then, the elastic constants of the structure were obtained as the ratios of effective stresses to the strains. It should be noted that the analyses in this paper were all in the elastic range without considering the non-linear characteristics of the materials and structures. Thus, linear finite element analyses were conducted in the calculations of the mechanical properties of the structure.

## 5. Results and Discussion

In this section, the results of the structural elastic constants obtained by the finite element analyses are plotted to verify those obtained by theoretical predictions. Then, the effects of geometric parameters α and β on the elastic properties are discussed, followed by suggestions for the structure in morphing applications. The minus signs on the right side of Equations (11) and (18) indicate the corresponding directions of the strains, so the absolute values of the theoretical strain ratios are used for plotting.

### 5.1. Elastic Modulus in the X-Direction

**Figure 7:**
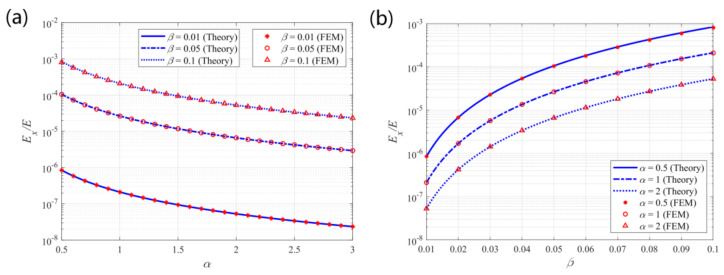
Theoretical predictions and finite element results for the effective elastic modulus *E_x_/E* vs. (**a**) α and (**b**) β.

Figure 7 shows the theoretical predictions and finite element results for the non-dimensional effective elastic modulus in the x-direction of the structure. The theoretical predictions are consistent with the finite element results. The modulus is in the range of 10^−8^ to 10^−3^, indicating a negative correlation with parameter α and a positive correlation with parameter β. Thus, a more expanded structure with slender beams would exhibit a low tensile modulus in the corresponding direction. Therefore, in morphing applications, large α and small β values are suggested to obtain a structure with low tensile stiffness, which is preferred for tension or compression.

### 5.2. Tensile–Shear Coupling Effect

**Figure 8:**
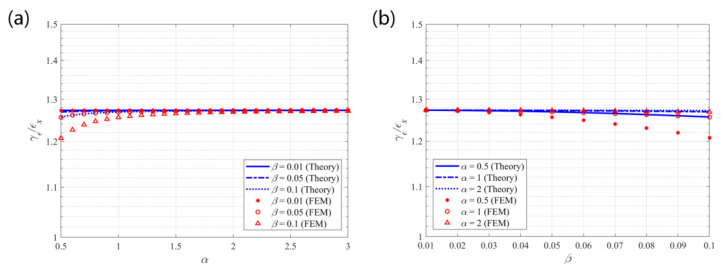
Theoretical predictions and finite element results for the coupling effect *γ_ε_/ε_x_* vs. (**a**) α; (**b**) β.

Figure 8 depicts the theoretical predictions and finite element results for the tensile–shear coupling effect under the tension in the x-direction of the structure. The theoretical results show good consistency with the finite element results. The largest error of 4.06% ((theoretical prediction—finite element result)/finite element result) is observed between the theoretical predictions and the finite element results at α = 0.5 and β = 0.1. Table 2 shows the errors between the theoretical and FE results of the shear strain to tensile strain ratio. The error decreases with the increase of α and decrease of β, in which case the constructing circular beams become slender. This discrepancy mainly results from the different beam models used in the theoretical and FE analyses. The internal shear forces in the beams are ignored in the theoretical derivations, while they are included in the FE analyses. Nevertheless, the theoretical results are credible with the maximum discrepancy lower than 5% compared to the FE results. It also provides the justification for the range selection of parameters for parametric study. Because larger values of parameter β would result in errors larger than 5% between theoretical predictions and FE results, in which case the theoretical formulae would be inaccurate in predicting the elastic properties of the structure. The shear strain to tensile strain ratio remains nearly constant with the variations of the parameters. Equation (11) also shows that β^2^ is a small quantity of a higher order for β ≤ 0.1 and has a limited contribution to the result. Then, the ratio is approximately 1.27 (≈4/π), indicating that the shear deformation per tensile strain is approximately 0.728°/%, which is obtained at (*γ_ε_/ε_x_*)∙180°/π/(100%).

### 5.3. Shear Modulus in the X–Y Plane

**Figure 9:**
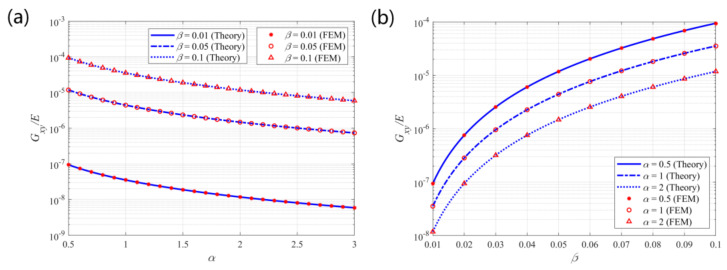
Theoretical predictions and finite element results for the effective shear modulus *G_xy_/E* vs. (**a**) α and (**b**) β.

Figure 9 shows the theoretical predictions and finite element results for the non-dimensional effective shear modulus in the x–y plane of the structure. The theoretical predictions are consistent with the finite element results. The shear modulus is in the range of 10^−8^ to 10^−4^, indicating a negative correlation with parameter α and a positive correlation with parameter β. Furthermore, a more expanded structure with slender beams shows lower in-plane shear modulus, which means that in morphing applications, large α and small β values are recommended for designing a structure with low shear stiffness that is easy for shear deformation.

### 5.4. Shear–Tensile Coupling Effects

**Figure 10:**
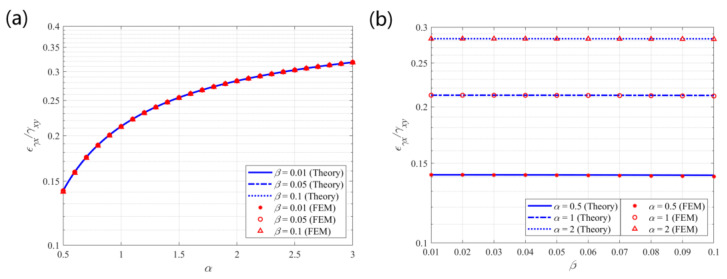
Theoretical predictions and finite element results for the coupling effect *ε_γx_/γ_xy_* vs. (**a**) α and (**b**) β.

Figure 10 shows the theoretical predictions and finite element results for the shear–tensile coupling effect in the x-direction under in-plane shear load. The theoretical predictions are consistent with the finite element results. The tensile strain to shear strain ratio is in the range of 0.1 to 0.4, indicating a positive correlation with parameter α, while remaining nearly constant with the variation of parameter β. The lowest value of the ratio is 0.14 at α = 0.5, while the largest value of the ratio is 0.32 at α = 3. Thus, the axial strain in the x-direction per shear strain is in the range of 0.24%/° to 0.56%/°, which is obtained at (*ε_γx_/γ_xy_*)∙π/180°/(100%).

**Figure 11:**
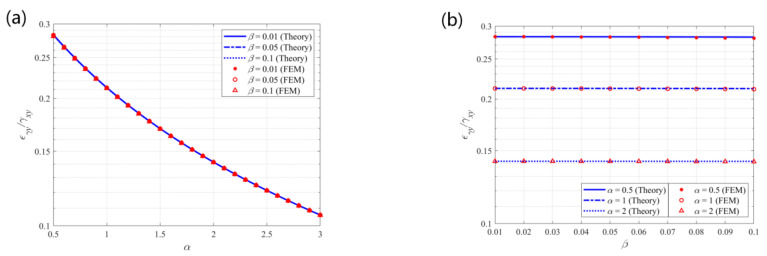
Theoretical predictions and finite element results for the coupling effect *ε_γy_/γ_xy_* vs. (**a**) α and (**b**) β.

Figure 11 shows the theoretical predictions and finite element results for the shear–tensile coupling effect in the y-direction under in-plane shear load. The theoretical predictions are consistent with the finite element results. The tensile strain to shear strain ratio is in the range of 0.1 to 0.3, indicating a negative correlation with parameter α, while exhibiting nearly no dependence on parameter β. The lowest value of the ratio is 0.11 at α = 3, while the largest value of the ratio is 0.28 at α = 0.5. Thus, the axial strain in the y-direction per shear strain is in the range of 0.19%/° to 0.49%/°, which is obtained at (*ε_γy_/γ_xy_*) π/180°/(100%).

In addition, Equation (18) shows that β^2^ is a small quantity of a higher order for β ≤ 0.1 and has little contribution to the strain ratios. Figure 10 and Figure 11 indicate that the coupling effects in the x- and y-directions are equal at α = 1, which can also be inferred from Equation (18). In this case, the axial strain in each direction per shear strain is 0.21 (=0.37%/°).

From Figure 8, Figure 10, and Figure 11 it is seen that the coupling effects of tensile and shear deformations are almost independent of geometric parameter β. The coupling effects are simultaneously affected by the topology of the structure and the geometric parameters. Once the structural topology is determined by the constructing circular beams, the coupling effects mainly depend on the weight of influence of the parameters. From Equations (11) and (17) it is seen that, within the default range of the parameters (0.5 ≤ α ≤ 3 and β ≤ 0.1), parameter α has greater impact on the coupling effects than parameter β. The coupling effects remain almost constant with the variation of parameter β.

The above results indicate that the chiral structure with circular beams shows low elastic and shear moduli and thus a good candidate for morphing applications. Large α and small β values can be employed to obtain a structure with a good morphing performance. In addition, the coupling effects should also be considered in the applications. Further studies can be carried out on the engineering applications of this chiral structure based on the structural elastic properties obtained in this study.

### 5.5. Failure Mechanism of the Structure

**Figure 12:**
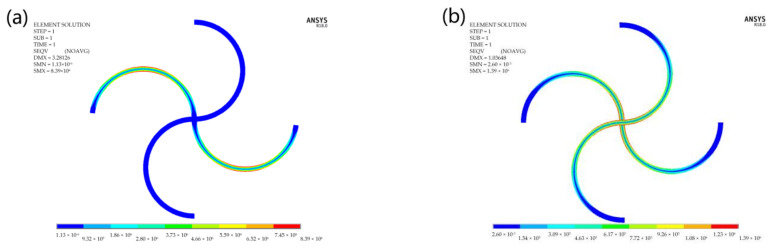
Stress distribution of the structure under in-plane: (**a**) axial load along the x-direction and (**b**) shear load.

Figure 12 shows the stress (Von Mises stress) distributions of the structure under in-plane axial and shear loads, respectively. It is seen that the stress only appears on the beam along the x-direction, while the beam along the y-direction does not bear any load, when the structure is undergoing in-plane axial load along the x-direction. The maximum stress occurs on the surface of the central point of each arc segment, where should be the most dangerous place that failure most likely occurs. When the structure is undergoing in-plane shear load, it is seen that the stress occurs on the beams along both in-plane directions. The maximum stress appears on the surface of the central connecting point of the beams. Thus, failure should firstly take place at the central connecting point of the unit cell with the increase of the in-plane shear load.

## 6. Conclusions

In this study, a chiral structure with circular beams is proposed. The feasible region of the geometric parameters is obtained by numerical analysis. Then, the in-plane elastic properties, including the elastic modulus, the shear modulus, and the coupling effects are studied through the combination of theoretical derivation and finite element analysis. The non-dimensional equivalent in-plane elastic modulus of the structure is in the range of 10^−8^ to 10^−3^, and the non-dimensional equivalent in-plane shear modulus is in the range of 10^−8^ to 10^−4^, indicating that the chiral structure has great potential for morphing applications. Both moduli show negative correlations with parameter α and positive correlations with parameter β, revealing that large α and small β values would result in a structure with a good morphing performance. The tensile-shear coupling ratio is nearly constant, that is, approximately 0.728 °/%. The shear-tensile coupling ratios in the x- and y-directions show opposite correlations with parameter α, and both are nearly unaffected by parameter β. The coupling effects should be considered in designing structures for engineering applications.

The novel structure proposed in this study shows extremely low in-plane moduli, making it good candidate for morphing application. It can be employed as core for sandwich morphing skin, or as flexible structures in variable camber wing. The axial and shear coupling effects also make it possible for compression-twist metamaterial application. Besides, the formulae of the elastic constants obtained in this study can provide fast predictions for the elastic properties of the structure in engineering applications. The analytical approach used in this study also can provide reference for studies of elastic properties of similar kind of chiral cellular structures.

## Figures and Tables

**Table 1:** Periodic and boundary conditions applied to the unit cell.

Conditions	Tensile Loading in the *x*-Direction	Shear Loading in the x–y Plane
Periodic conditions	$\begin{array}{c} u_{x}\left(B\right)=u_{x}\left(D\right)\\ u_{y}\left(B\right)=u_{y}\left(D\right)\\ \theta_{z}\left(A\right)=\theta_{z}\left(C\right)\\ \theta_{z}\left(B\right)=\theta_{z}\left(D\right) \end{array}$	$\begin{array}{c} u_{y}\left(A\right)=u_{y}\left(C\right)\\ \theta_{z}\left(A\right)=\theta_{z}\left(C\right)\\ \theta_{z}\left(B\right)=\theta_{z}\left(D\right) \end{array}$
Boundary conditions	$\begin{array}{c} u_{x}\left(A\right)=0\\ u_{y}\left(A\right)=0\\ u_{x}\left(C\right)=2H\varepsilon_{x} \end{array}$	$\begin{array}{c} u_{x}\left(B\right)=0\\ u_{y}\left(B\right)=0\\ u_{x}\left(D\right)=2L\gamma_{xy} \end{array}$
	z-direction~SYMM for all nodes	

**Table 2:** Errors between theoretical and FE results.

Parameters	β = 0.01					
	α = 0.5	α = 0.6	α = 0.7	α = 0.8	α = 0.9	α = 1
Theoretical result	1.2731	1.2731	1.2732	1.2732	1.2732	1.2732
FE result	1.2725	1.2728	1.2729	1.2730	1.2730	1.2731
Error	4.12 × 10^−4^	2.86 × 10^−4^	2.10 × 10^−4^	1.61 × 10^−4^	1.27 × 10^−4^	1.03 × 10^−4^
**Parameters**	**β = 0.05**					
	**α = 0.5**	**α = 0.6**	**α = 0.7**	**α = 0.8**	**α = 0.9**	**α = 1**
Theoretical result	1.2690	1.2703	1.2711	1.2716	1.2719	1.2722
FE result	1.2561	1.2613	1.2644	1.2665	1.2679	1.2689
Error	1.03 × 10^−2^	0.71 × 10^−2^	0.52 × 10^−2^	0.40 × 10^−2^	0.32 × 10^−2^	0.26 × 10^−2^
**Parameters**	**β = 0.1**					
	**α = 0.5**	**α = 0.6**	**α = 0.7**	**α = 0.8**	**α = 0.9**	**α = 1**
Theoretical result	1.2565	1.2616	1.2646	1.2666	1.2680	1.2690
FE result	1.2074	1.2268	1.2388	1.2467	1.2522	1.2561
Error	4.06 × 10^−2^	2.83 × 10^−2^	2.09 × 10^−2^	1.60 × 10^−2^	1.27 × 10^−2^	1.03 × 10^−2^

## Data Availability

Data is contained within the article.
